# Response to letter to the editor regarding “Does the 5-2-1 criteria identify patients with advanced Parkinson’s disease? Real-world screening accuracy and burden of 5-2-1-positive patients in 7 countries”

**DOI:** 10.1186/s12883-024-03691-3

**Published:** 2024-07-30

**Authors:** Angelo Antonini, K. Ray Chaudhuri, Josefa Domingos, Joohi Jimenez-Shahed, Jack Wright, Connie H. Yan, Ali Alobaidi, Lars Bergmann, Koray Onuk, Linda Harmer, Irene A. Malaty

**Affiliations:** 1 Parkinson and Movement Disorders Unit, Department of Neuroscience, University of Padua, Padua, Italy; 2 Parkinson Foundation Centre of Excellence, King’s College Hospital and King’s College, London, UK; 3 European Parkinson’s Disease Association (EPDA), Sevenoaks, UK; 4 Grupo de patologia médica, nutrição e exercício clínico (PaMNEC) do CiiEM, Lisbon, Portugal; 5 Department of Neurology, Icahn School of Medicine at Mount Sinai, New York, NY USA; 6 Adelphi Real World, Bollington, UK; 7 AbbVie Inc, North Chicago, IL USA; 8 Fixel Institute for Neurological Diseases, University of Florida, Gainesville, FL USA

**Keywords:** Advanced Parkinson’s disease, Device-aided therapies, Patient identification, 5-2-1 criteria, Screening performance

## Abstract

The 5-2-1 criteria was developed to facilitate the identification and referral of patients with Parkinson’s Disease (PD) inadequately controlled by oral medications. The criterion was not developed to screen patients with PD for device-aided therapy eligibility. The robust design and validation of the 5-2-1 criteria minimizes over or inappropriate referrals, and supports physicians in the timely identification of patients with PD who may warrant further evaluation for treatment optimization. This response letter clarifies concerns raised by Moes et al.

Dear Editor,

Thank you for the opportunity to respond to the issues raised by Moes et al., and to provide clarifications relating to their concerns regarding our recently published article, entitled “*Does the 5-2-1 criteria identify patients with advanced Parkinson’s disease? Real-world screening accuracy and burden of 5-2-1-positive patients in 7 countries*” [1].

As noted by Moes et al., this study was conducted in a large cohort of 4714 patients, is multicentred across 7 countries, and leverages data derived from the Adelphi Parkinson’s Disease Specific Programme (DSP)™, a secondary data source which uses previously published and validated methodology to capture evidence reflecting current clinical practice [2–4], and provides a large and robust real-world sample to further validate the 5-2-1 criteria.

As stated in the response by Moes et al., the 5-2-1 criteria was developed based on expert opinion to support physician’s identification of patients with advanced Parkinson’s Disease (PD) who are inadequately controlled by oral medications. An important clarification, this criteria was not intended for supporting referral of patients for device aided therapy (DAT) although treatment optimization in this population includes DAT. The items of the 5-2-1 criteria are key clinical indicators of suspected advanced PD proposed and endorsed by an international Delphi-panel of Movement Disorder Specialist [5]. Considering the intent of the 5-2-1 criteria is to ensure timely identification of advanced PD patients and, the assessment of screening accuracy using Area Under the Curve (AUC) and validation amongst an international group of neurologists and MDS is fit-for-purpose.

With respect to the comment relating to the prevalence of advanced PD observed in our study (14.9%), this was likely a consequence of the survey methodology sampling from the consulting population rather than taking a random sample from the PD population. Patients with more advanced PD may consult more frequently and were therefore more likely to be recruited via the DSP survey methodology, and this sample of the consulting population may be more reflective of a neurologist’s day to day experience than a truly random sample of patients with PD. Furthermore, it should be noted that the prevalence of advanced PD in our study aligns with those previously summarised in the literature [6].

Advanced PD is a subjective term due to the lack of standardized diagnostics. Therefore, by determining the overlap of the subjective and objective definitions we show that the readily available, and objective 5-2-1 criteria largely mirrors the considered opinions of the real-world practicing neurologists (best available proxy of the gold standard criterion) when arriving at their subjective conclusion. Furthermore, the reference test was performed by each respondent neurologist in isolation, for their specific portion of the patient sample (12 patients per neurologist). In total, 563 neurologists provided data for 6241 of their consulting patients with PD, of whom 4714 met the inclusion criteria for this analysis. The level of experience of the recruited neurologists will have varied, with the exception that they were required to see on average three or more patients with PD per week. While this reference is a subjective measure that will have varied on a physician-to-physician basis, we believe this is the best available reference in large sample of patients and is reflective of real-world clinical practice.

Additionally, we tested the 5-2-1 criteria against patient Hoehn & Yahr (H&Y) stages as an alternative reference. This alternative analysis yielded broadly comparable results to the original analysis, and further supports the validity of the screening measure. It should be noted that as this study utilised a secondary data source, these were not design decisions, but rather features of the existing data.

We did not discuss the predictive value of the 5-2-1 criteria in the original study as the focus of our research was on the screening accuracy. The sensitivity and specificity calculations reported by Moes et al. are correct for the unadjusted data, however these were not reported in the original manuscript because we wanted to focus on the results of the adjusted model. The AUC was our preferred measure of screening accuracy because this measure balances both sensitivity and specificity [7–9]. The screening accuracy evaluation of 5-2-1 are presented as adjusted estimates in Table 1 below, accounting for age, sex, time since diagnosis, Charlson Comorbidity Index, and country, in comparison to the unadjusted data as calculated and discussed by Moes et al. Use of adjustment was deemed appropriate in the context of the multi-country, secondary dataset.

**Table 1:** Adjusted and unadjusted screening accuracy of the 5-2-1 criteria in identifying patients with advanced Parkinson’s disease

Indicator		Physician judgement			Screening accuracy measure	Unadjusted	Adjusted model ^a^
		Not APD	APD				
5-2-1 screening criteria					Correct classification (%) ^b^	75.7	88.1
					Sensitivity (%)	78.6	41.9
Negative		3018	150		Specificity (%)	75.2	96.2
					PPV (%) ^c^	35.7	65.9
Positive		994	552		NPV (%) ^d^	95.3	90.4
					AUC ^e^	-	0.89

^a^ Regressions adjusted for age, sex, time since diagnosis, Charlson Comorbidity Index, and country.

^b^ Correct classification is the percentage of patients correctly classified per 5-2-1 criteria (sum of true positive and true negatives divided by total number of patients).

^c^ PPV is the percentage of true positives among patients with a positive screening result (number of true positives divided by the sum of true and false positives).

^d^ NPV is the percentage of true negatives among patients with a negative screening result (number of true negatives divided by the sum of true and false negatives).

^e^ AUC is a screening accuracy measure that balances sensitivity and specificity.

*APD* Advanced Parkinson’s disease, *AUC* Area under the curve, *PPV* Positive predictive value, *NPV* Negative predictive value.

Following regression, the predicted probabilities were derived from the model and patients are classified as Advanced or Not Advanced according to a cut-point of 0.5 (it was felt this was most intuitive in a predictive regression setting of two categories, e.g. more likely to belong to one category than another), yielding a concordance of 88.1%. The 5-2-1 criteria demonstrated moderate accuracy (AUC of 0.89) in identifying advanced PD patients based on widely accepted thresholds. The adjusted model yielded a higher specificity of 96.2%, and positive predictive value (PPV) of 65.9%, resulting in a lower rate of false positives. The adjusted sensitivity was lower at 41.9%, however the negative predictive value (NPV) remained high at 90.4% (a previous version of this correspondence associated this with a low rate of false negatives, which we acknowledge was incorrectly described). It’s worth noting that the screening accuracy is also reflective of reference which is based on neurologist judgement. Therefore, heterogeneity in real-world assessment of advanced PD could have impacted the observed accuracy of the 5-2-1 criteria in this study.

To reiterate, the 5-2-1 criteria is intended to facilitate identifications of patients with advanced PD including those who may not be adequately controlled by oral medications. As a screening aid, the 5-2-1 criteria can only help identify individuals who may benefit from further intervention or alternate treatment options, which may include specialist input. As noted, the criteria does not encompass many symptoms such as cognition or autonomic function but it focuses on the characteristics of response to levodopa therapy including number of daily intakes. Therefore, patients screened by the 5-2-1 criteria should be further evaluated by the neurologist before consideration of treatment optimization and/or determine if referral to a specialized institution is warranted. Additionally, the careful validation and design of the 5-2-1 criteria minimizes over or inappropriate referrals.

The 5-2-1 criteria is not intended to identify patients for DAT eligibility, nor is an indication to refer patients for DAT which requires more accurate clinical evaluation or dedicated screening tools, such as the MANAGE-PD [10].

Physicians can use the 5-2-1 criteria to quickly assess patients’ clinical status, identify those who are sub-optimally controlled on current therapy, and take timely action to consider treatment changes or make referrals to specialized centres. We understand the concern about the risk of excessive referral, but believe that burden on referral networks should not be a reason to prevent patients from being referred when warranted. We should all be aware that in most countries, under-referral is a greater risk, potentially contributing to suboptimal management including underutilization of advanced therapies. Finally, we believe it is in the interest of patients being referred to a specialized centre in case of inadequately controlled symptomatology and implementation of easily applicable 5-2-1 criteria should encourage more efficient interaction in regional health networks as suggested by recent MDS-ES guidelines for invasive therapies in PD [11, 12].

## Data Availability

No datasets were generated or analysed during the current study.

## Abbreviations

AUC: Area under the curve
DAT: Device aided therapy
DSP™: Disease Specific Programme™
MDS: Movement Disorder Specialist
NPV: Negative predictive value
PD: Parkinson’s Disease
PPV: Positive predictive value
